# Human Epididymis Protein 4 in Transcatheter Aortic Valve Implantation: Diagnostic and Prognostic Value

**DOI:** 10.1016/j.jacadv.2025.101722

**Published:** 2025-04-25

**Authors:** Carlos Giuliani, Antonela Zanuttini, Jorge Nuche, Julio I. Farjat Pasos, Jérémy Bernard, Tastet Lionel, Simon Jacob, Rami Abu-Alhayja’a, Jonathan Beaudoin, Nancy Côté, Robert DeLarochellière, Jean-Michel Paradis, Marie-Annick Clavel, Benoit J. Arsenault, Josep Rodés-Cabau, Philippe Pibarot, Sébastien Hecht

**Affiliations:** a Centre de recherche de l’Institut universitaire de cardiologie et de pneumologie de Québec (IUCPQ), Québec City, Québec, Canada; b Faculté de médecine, Université Laval, Québec City, Québec, Canada

**Keywords:** aortic stenosis, transcatheter aortic valve implantation, human epididymis protein 4, heart failure, myocardial fibrosis

## Abstract

**Background:**

The utility of the human epididymis protein 4 (HE4) in patients undergoing transcatheter aortic valve implantation (TAVI) has not been established yet.

**Objectives:**

The present study aimed at examining the prognostic value of HE4 in patients undergoing TAVI.

**Methods:**

In this prospective study, the prognostic value of HE4 to predict adverse clinical events was evaluated in 362 patients who underwent TAVI. The association between HE4 and diffuse myocardial fibrosis was also assessed using T1 mapping on cardiac magnetic resonance in a subgroup of 43 patients.

**Results:**

During a median follow-up of 2.5 (IQR: 1.9-3.2) years, 34/362 (9.4%) patients were rehospitalized for heart failure, 99/362 (27.3%) died, and 113/362 (31.2%) met the composite endpoint of rehospitalization for heart failure or all-cause mortality. In multivariable Cox regression analyses, patients with higher HE4 serum levels (ie, HE4 ≥130 pmol/L) vs lower serum levels (ie, HE4 <130 pmol/L) had increased risk of all-cause mortality (adjusted HR: 3.26 [95% CI: 2.04-5.20], *P* < 0.001), and of the composite endpoint (adjusted HR: 2.48 [95% CI: 1.64-3.74], *P* < 0.001) following TAVI, respectively. Patients with higher HE4 serum levels had higher median native T1 mapping values (1,278 [95% CI: 1,239-1,280] ms vs 1,352 [95% CI: 1,303-1,376] ms, *P* < 0.001) at 1 to 3 months following the procedure.

**Conclusions:**

Elevated HE4 serum levels are associated with diffuse myocardial fibrosis and increased risk of adverse clinical events following TAVI. This promising blood biomarker may be helpful to enhance risk stratification in patients undergoing TAVI.

Transcatheter aortic valve implantation (TAVI) is a valuable alternative to surgical aortic valve replacement for the treatment of severe aortic stenosis (AS), regardless of the surgical risk.^1^, ^2^, ^3^ However, TAVI is futile in a substantial proportion of patients who die shortly after the procedure or do not experience any improvement in quality of life.^4^, ^5^, ^6^, ^7^ The European System for Cardiac Operative Risk Evaluation II (EuroSCORE II) score and Society of Thoracic Surgeons (STS) score are extensively used for risk prediction in patients undergoing TAVI. However, both scores were developed and validated in conventional cardiac surgery population and tend to systematically overestimate procedural mortality in patients undergoing TAVI. TAVI-specific risk scores have been developed to improve risk stratification and prognosis, but none of them are optimal and have been adopted in the clinical setting.^8^, ^9^, ^10^, ^11^, ^12^ There are relatively few studies on the usefulness of blood biomarkers for risk stratification after TAVI. Over the last decade, there has been a growing interest in blood biomarkers to improve risk stratification in patients with underlying cardiac conditions. We previously reported that a multimarker approach provides incremental value over clinical and echocardiographic parameters to predict mortality and treatment futility following TAVI or surgical valve replacement.^13^, ^14^, ^15^

The human epididymis protein 4 (HE4) is a four-disulfide core domain 2 acidic protein that is expressed in a variety of bodily organs, including the kidney, digestive tract, and respiratory system.^16^^,^^17^ It is also known as a protease inhibitor that suppresses the activities of multiple proteases, such as serine proteases and matrix metalloproteinases.^18^ This is a promising blood biomarker associated with renal and cardiac fibrosis, increased rates of heart failure (HF) and cardiac events.^15^^,^^19^, ^20^, ^21^ However, the value of this biomarker for risk classification in the context of TAVI and its association with myocardial fibrosis have not been explored. The objective of this study was thus to investigate the prognostic value and association with myocardial fibrosis of HE4 in patients with severe AS undergoing TAVI.

## Methods

### Population

A total of 362 consecutive patients were prospectively enrolled at Québec Heart and Lung Institute in the TAVI-B study (Transcatheter Aortic Valve Implantation Biomarkers) from January 2017 to August 2020. Patients were included if they had symptomatic severe AS and were candidates for TAVI after Heart Team discussions based on clinical recommendations.^22^^,^^23^ Exclusion criteria included patients who previously had multiple surgical interventions on the aortic valve. The study received approval from the Institutional Review Board committee of the participating center, and the patients provided written informed consent. Clinical and echocardiographic variables were gathered at preintervention, discharge, 1 to 3 months, and at 1-year post-TAVI. Additionally, a subgroup of 43 patients underwent cardiac magnetic resonance (CMR) at baseline and within 3 months following TAVI procedure.

### Laboratory measurements

Blood samples were collected before TAVI procedure and stored at −80 C. The analyses of the blood biomarkers N-terminal B-type natriuretic peptide (NT-proBNP) and HE4 were performed using enzyme-linked immunosorbent assay kits approved for clinical use and commercialized by Roche Diagnostics. HE4 serum level was considered elevated in a given patient if equal or above the optimal prognostic value of (≥130 pmol/L), as determined using receiver-operating characteristic (ROC) curve analysis regarding all-cause mortality. The ratio between measured serum NT-proBNP levels and maximal normal NT-proBNP levels for age and sex (NT-proBNP ratio) was calculated for each patient.

### Doppler echocardiography

As previously described,^15^ the mean transvalvular pressure gradient was measured with the simplified Bernoulli formula.^24^ The stroke volume indexed was calculated by multiplying the cross-sectional area of the left ventricular outflow tract with the velocity-time integral measured below the valve and divided by body surface area.^24^ Aortic valve area was calculated using the continuity equation. Aortic, mitral, and tricuspid valve regurgitations were assessed using a multiparameter integrative approach as previously described.^25^ LV systolic function was assessed by the measurement of left ventricular ejection fraction (LVEF) using the biplane Simpson method.^26^ All other measurements were performed according to the European Association of Cardiovascular Imaging and American Society of Echocardiography guidelines.^26^ Analyses of echocardiogram were performed using the TomTec Imaging Platform (V.4.6, Image Arena TM) software.

### Cardiovascular magnetic resonance

Imaging was performed on either a 3-T Philips Ingenia scanner (software versions R5.1.9 and R5.3.0) using the integrated two-channel body coil for excitation and a 28-channel body array surface coil with cardiac capability for signal reception (Philips Healthcare). Focal replacement fibrosis was assessed using late gadolinium enhancement (LGE) and diffuse myocardial fibrosis by expansion of the extracellular volume using T1 mapping. LGE was performed 15 minutes after administration of 0.1 mmol/kg of gadobutrol (Gadovist, Bayer Pharma AG). The presence of mid-wall focal myocardial fibrosis was determined qualitatively by one experienced operator, and its distribution was recorded.^27^^,^^28^ T1 mapping was performed preinjection and postinjection (after a 15-minute delay) using the Modified Look-Locker approach.^28^^,^^29^

### Study endpoints

The primary study endpoints were: 1) all-cause mortality; and 2) a composite of rehospitalization for HF and all-cause mortality. The secondary endpoints were: 1) diffuse myocardial fibrosis as defined as a native T1 mapping median value >1,280 ms; 2) focal myocardial fibrosis as defined as LGE positive; 3) rehospitalization for HF; and 4) treatment futility defined as a composite of NYHA functional class ≥III, rehospitalization for HF, or all-cause mortality at 1 year. Mortality was obtained from the Central Québec Institute of Statistics database.

### Statistical analyses

Continuous variables were first tested for normality by the Shapiro-Wilk test or the Kolmogorov-Smirnov test and expressed as median (IQR) or mean ± SD, as appropriate. Univariable Cox proportional hazards regression analysis was used to evaluate the association between serum levels of HE4 with all-cause mortality. The optimal prognostic cutoff value for HE4 (130 pmol/L) to predict all-cause mortality was initially determined using ROC curve analysis, with the Youden index identifying the optimal cutoff. To confirm this threshold, a maximally selected rank test was subsequently performed (Supplemental Figure 1). Then, the cohort was divided into 2 groups according to HE4 serum levels (<130 or ≥130 pmol/L) at baseline. Continuous variables were compared using Mann-Whitney tests, and categorical variables were compared using the chi-square or Fisher exact test and expressed in number of patients with percentages. Cumulative incidence of all-cause mortality, rehospitalization for HF, and the composite endpoint of rehospitalization for HF and all-cause mortality according to HE4 serum levels were assessed using Kaplan-Meier analysis and compared using the log-rank test.

To account for potential confounders, univariate Cox regression analyses were performed for all-cause mortality, the composite endpoint of HF rehospitalization and all-cause mortality, and treatment futility. Prespecified variables with a significance level of *P* < 0.05 in univariable Cox regression analyses were subsequently included in multivariable Cox proportional hazards regression models. The absence of collinearity between independent variables in each multivariable model was verified through variance inflation factor. For all-cause mortality, analyses were adjusted for diabetes, coronary artery disease (CAD), chronic obstructive pulmonary disease (COPD), atrial fibrillation, LVEF <50%, and NT-ProBNP ratio ≥3. For the composite endpoint of HF rehospitalization and all-cause mortality, analyses were adjusted for diabetes, atrial fibrillation, CAD, NT-ProBNP ratio ≥3, and LVEF <50%. For treatment futility, analyses were adjusted for hypertension, diabetes, COPD, NT-ProBNP ratio ≥3, and LVEF <50%. Additionally, further multivariable Cox regression analyses were performed to include the STS score in the multivariable model, along with HE4 and NT-ProBNP ratio. The results are presented as HR with 95% CIs and proportional hazards assumption was confirmed using scaled Schoenfeld residuals. To account for competing risks, a Fine-Gray subdistribution hazards model was used for rehospitalization for HF, adjusted for atrial fibrillation and NT-ProBNP ratio ≥3. The prognostic value of HE4, NT-ProBNP, and STS score regarding all-cause mortality was assessed using ROC analysis. Harrell's C-index and the likelihood ratio test were used to evaluate the incremental prognostic value of HE4 over a nested model (diabetes, COPD, atrial fibrillation, CAD, LVEF<50%, and NT-ProBNP ratio>3 for all-cause mortality. Decision curve analysis to assess the clinical utility of HE4 ≥130 in addition to clinical covariates. Two models were compared: one including only clinical covariates (diabetes, COPD, atrial fibrillation, CAD, LVEF <50%, and NT-proBNP ≥3) and another incorporating HE4. The optimal diagnostic threshold of HE4 (130 pmol/L) to identify diffuse myocardial fibrosis was assessed in a subgroup analysis of 43 patients using ROC curve analysis, with the Youden index used to identify the optimal diagnostic cutoff point. Spearman correlation was computed to examine the relationship between HE4 serum levels and native T1 mapping by CMR and NT-proBNP. The association between baseline characteristics and native T1 mapping was assessed using logistic regression analysis. ORs and 95% CIs were reported for each group. All tests were two-sided and *P* values <0.05 were considered statistically significant. Statistical analyses were performed using SPSS version 25.0 (IBM Corporation) and STATA version 14.0 (StataCorp).

## Results

### Baseline characteristics of study population

Baseline characteristics of the study population according to serum levels of HE4 are shown in Table 1. Among the 362 patients included in the present study, 219/362 (60.5%) had serum levels <130 pmol/L and 143/362 (39.5%) had serum levels ≥130 pmol/L. Patients with low HE4 serum levels, patients with higher serum levels of HE4 were older, more often men, had a higher prevalence of comorbidities, higher surgical risk scores, and higher serum levels of NT-proBNP (*P* < 0.001) and creatinine (*P* < 0.001) (Table 1). Of note, there was a significant correlation between NT-proBNP and HE4 serum levels (r = 0.51, *P* < 0.001). LVEF, mean transvalvular pressure gradient, and stroke volume indexed were lower in patients with higher serum levels of HE4 (*P* < 0.001). The frequencies of significant (ie, ≥moderate) mitral and tricuspid regurgitation were also higher (*P* < 0.001) (Table 2). The prevalence of symptoms according to HE4 serum levels is represented in Supplemental Figure 2.

**Table 1:** Baseline Characteristics According to HE4 Serum Level

	All Patients (N = 362)	HE4 <130 pmol/L (n = 219, 60.5%)	HE4 ≥130 pmol/L (n = 143, 39.5%)	*P* Value
Clinical data				
Age, y	79.6 ± 8.2	78.4 ± 8.1	81.4 ± 8.1	**<0.001**
Male	208 (57.3)	111 (51.1)	95 (66.4)	**0.040**
Body mass index, kg/m^2^	28.1 ± 6.6	28.8 ± 7.2	28 ± 6.3	0.439
Hypertension	322 (88.7)	192 (87.7)	130 (90.9)	0.337
Dyslipidemia	304 (83.7)	181 (81.4)	123 (86.6)	0.271
Diabetes mellitus	132 (36.4)	70 (31.8)	62 (43.7)	**0.022**
Smoking history	92 (25.5)	51 (23.4)	40 (28.4)	0.510
Cancer in remission	59 (16.3)	34 (14.5)	25 (17.6)	0.735
Cancer active	13 (6.3)	7 (3.2)	6 (4.2)	0.735
CHF	118 (32.7)	54 (24.7)	63 (44.7)	**<0.001**
Previous MI	37 (10.2)	20 (9.2)	17 (12.0)	0.393
COPD	80 (22.1)	44 (20.0)	36 (25.4)	0.231
History of AF	125 (34.7)	64 (29.5)	60 (42.3)	**0.013**
Pacemaker	60 (16.3)	30 (13.6)	29 (20.4)	0.088
Prior LBBB	28 (7.7)	14 (6.4)	14 (9.8)	0.181
CAD	218 (60.2)	124 (56.6)	94 (66.2)	0.069
History of CABG	93 (25.6)	55 (25.0)	38 (26.8)	0.708
History of PCI	153 (42.1)	86 (39.1)	67 (47.2)	0.128
Cerebrovascular disease	31 (8.5)	19 (8.6)	11 (7.7)	0.764
History of TIA	36 (9.9)	20 (9.1)	15 (10.6)	0.643
Peripheral vascular disease	81 (22.3)	40 (18.2)	41 (28.9)	**0.017**
Renal failure	174 (48.5)	67 (30.9)	107 (75.9)	**<0.001**
Euro SCORE II	4.0 (2.3-7.7)	2.9 (1.8-5.0)	6.1 (3.4-11.1)	**<0.001**
STS score	3.9 (2.7-6.0)	3.2 (2.3-4.5)	5.4 (3.9-8.4)	**<0.001**
NYHA functional class ≥III	235 (64.7)	135 (61.4)	100 (69.9)	0.105
Laboratory data				
HE4, pmol/L	114.9 (79.9-162.9)	87.8 (67.9-108.9)	184.8 (151.5-275.5)	–
NT-ProBNP, pg/mL	1,373.0 (492.5-3,641.0)	836.9 (386.6-2,010.0)	2,975.5 (933.2-8,018.0)	**<0.001**
NT-proBNP ratio	2.8 (1.8-6.9)	2.0 (0.7-4.0)	5.7 (1.8-18.4)	**<0.001**
Creatinine, mmol/L	89 (70-117.5)	74.0 (66.0-90.0)	120.5 (101.0-152.5)	**<0.001**
eGFR, mL/min	51.9 (40.4-70.1)	60.7 (50.3-79.5)	40.5 (30.6-51.1)	**<0.001**

Values are mean ± SD, n (%), or median (IQR). *P* values refer to comparison between group of number biomarkers elevated. *P* values <0.05 were considered statistically significant.

AF = atrial fibrillation; CABG = coronary artery bypass grafting; CAD = coronary artery disease; CHF = congestive heart failure; COPD = chronic obstructive pulmonary disease; eGFR = estimate glomerular filtrate rate; EuroSCORE II = European System for Cardiac Operative Risk Evaluation II; HE4 = human epididymis protein 4; LBBB = left bundle branch block; MI = myocardial infarction; NYHA = New York Heart Association functional class; NT-proBNP = N-terminal B-type natriuretic peptide; PCI = percutaneous coronary intervention; TIA = transient ischemic attack; STS = Society of Thoracic Surgeons.

**Table 2:** Baseline Echocardiographic Characteristics According to HE4 Serum Level

	All Patients (N = 362)	HE4 <130 pmol/L (n = 219, 60.5%)	HE4 ≥130 pmol/L (n = 143, 39.5%)	*P* Value
LVEF, %	53.6 ± 11.8	56.1 ± 9.7	50.0 ± 13.6	**<0.001**
MG, mm Hg	43.2 ± 17.5	45.3 ± 17.3	40.3 ± 17.2	**0.006**
EOA, cm^2^	0.71 ± 0.25	0.70 ± 0.19	0.72 ± 0.32	0.556
EOAi, cm^2^/m^2^	0.38 ± 0.11	0.38 ± 0.10	0.37 ± 0.10	0.167
SVi, mL/m^2^	36.49 ± 8.7	36.8 ± 7.8	34.7 ± 9.5	**0.045**
SVi ≤35 mL/m^2^	80 (22.0)	45 (46.4)	31 (26.7)	0.059
Classical LF-LG, n (%)	143 (39.5)	81 (39.3)	62 (48.1)	0.115
Paradoxical LF-LG, n (%)	62 (17.1)	37 (16.9)	25 (17.5)	0.885
AR ≥moderate, n (%)	60 (16.5)	33 (15.1)	26 (18.2)	0.433
MR ≥moderate, n (%)	84 (23.1)	38 (17.4)	46 (32.2)	**0.001**
TR ≥moderate, n (%)	42 (18.2)	16 (11.3)	26 (31.7)	**<0.001**
PAPs ≥50 mm Hg, n (%)	141 (38.4)	87 (39.7)	54 (37.8)	0.708

Values are mean ± SD or n (%). *P* values <0.05 were considered statistically significant.

AR = aortic regurgitation; EOA = effective orifice area; LF-LG = low-flow low-gradient; LVEF= left ventricular ejection fraction; MR = mitral regurgitation; MG = mean transvalvular pressure gradient; PAPs = systolic pulmonary artery pressure; SVi = stroke volume indexed; TR = tricuspid regurgitation; other abbreviation as in Table 1.

### Procedural and short-term outcomes

Procedural data outcomes following the procedure are presented in Supplemental Tables 1 and 2. In comparison with patients with lower serum levels of HE4, patients with higher serum levels had significantly higher rates of dialysis, new onset of atrial fibrillation, length of hospital stay, as well as higher rates of 30 days, 1-year all-cause mortality, cardiovascular mortality, and rehospitalization for HF (*P* < 0.001).

### Prognostic value of HE4 serum levels

During a median follow-up of 2.5 (IQR: 1.9-3.2) years, 34/362 (9.4%) patients were rehospitalized for HF, 99/362 (27.3%) died, 113/362 (31.2%) met the composite endpoint of all-cause mortality or rehospitalization for HF and 70/362 (19.3%) met the composite of treatment futility.

**Figure 1:**
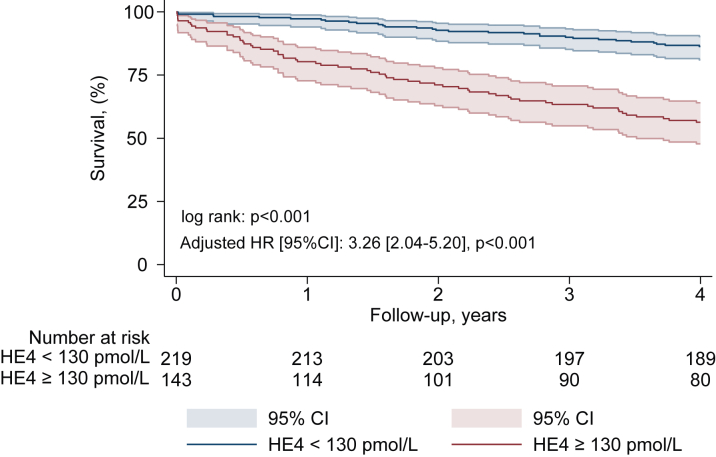
All-Cause Mortality According to HE4 Serum Level Kaplan-Meier survival estimates according to serum level of HE4: <130 pmol/L (blue) and ≥130 pmol/L (red) per patients prior to transcatheter aortic valve implantation. Adjusted HR for diabetes, coronary artery disease, COPD, atrial fibrillation, LVEF <50%, and NT-ProBNP ratio ≥3. COPD = chronic obstructive pulmonary disease; HE4 = human epididymis protein 4; LVEF= left ventricular ejection fraction; NT-proBNP = N-terminal B-type natriuretic peptides.

At 4 years, Kaplan-Meier survival estimates were 85%, in patients with high and low serum levels of HE4, respectively, log-rank, *P* < 0.001, Figure 1. The areas under the curve (AUC) to predict all-cause mortality at 4 years for STS score, NT-ProBNP and HE4 were AUC: 0.68 [95% CI: 0.62-0.74] *P* < 0.001, 0.64 [95% CI: 0.58-0.71] *P* < 0.001, and 0.72 [95% CI: 0.65-0.78], *P* < 0.001, respectively (Central Illustration, Supplemental Table 3).

**Central Illustration:**
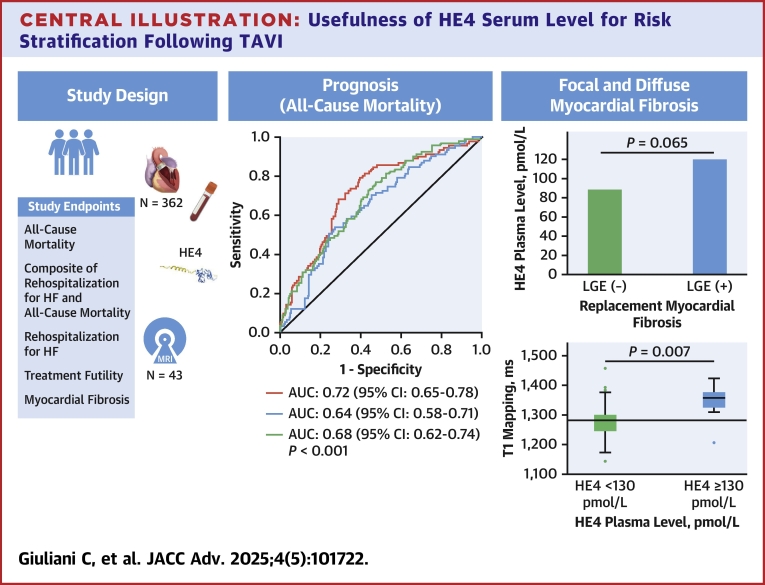
Usefulness of HE4 Serum Level for Risk Stratification Following TAVI ROC curve analysis assessing the prognostic value of HE4 (red line), STS score (green line), and NT-proBNP (blue line) to predict all-cause mortality. AUC = area under the curve; LGE = late gadolinium enhancement; TAVI = transcatheter aortic valve implantation; ROC = receiver-operating characteristic; STS = Society of Thoracic Surgeons; other abbreviations as in Figures 1 and 2.

In univariate analyses, a serum level of HE4 ≥130 pmol/L was associated with a higher risk of all-cause mortality (HR: 3.45 [95% CI: 2.14-5.57], *P* < 0.001) (Supplemental Table 3), rehospitalization for HF (sHR: 2.62 [95% CI: 1.31-5.25], *P* < 0.007) (Supplemental Table 4), the composite endpoint of rehospitalization for HF and all-cause mortality (HR: 3.15 [95% CI: 2.15-4.62], *P* < 0.001) (Supplemental Table 5) and treatment futility (HR: 3.59 [95% CI: 2.18-5.91], *P* < 0.001) (Supplemental Table 6).

**Figure 2:**
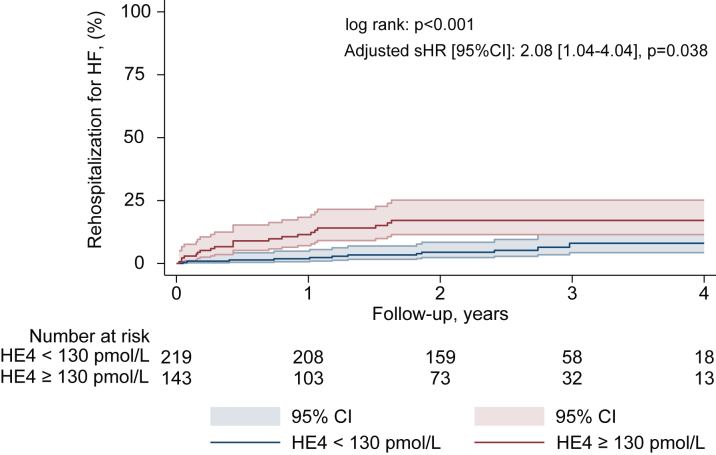
Rehospitalization for Heart Failure According to HE4 Serum Level Kaplan-Meier cumulative estimates of rehospitalization for heart failure according to HE4 serum levels. Curves represent rehospitalization for heart failure according to serum level of HE4: <130 pmol/L (blue) and ≥130 pmol/L (red). Adjusted subdistribution HR for atrial fibrillation and NT-proBNP ratio ≥3. HF = heart failure; other abbreviations as in Figure 1.

**Figure 3:**
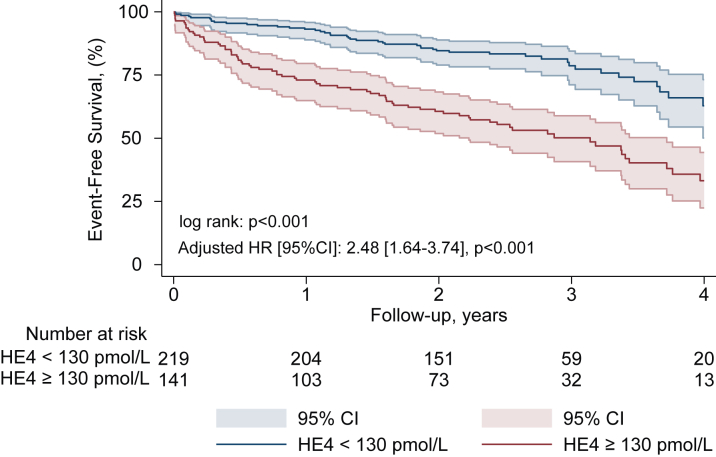
Composite Endpoint Rehospitalization for Heart Failure and All-Cause Mortality According to HE4 Serum Level Kaplan-Meier event-free survival estimates according to HE4 serum level. Curves represent survival according to serum level of HE4: <130 pmol/L (blue) and ≥130 pmol/L (red). Adjusted HR for diabetes, atrial fibrillation, coronary artery disease, NT-proBNP ratio ≥3, and LVEF <50%. Abbreviations as in Figure 1.

In multivariable analyses, a serum level of HE4 ≥130 pmol/L was independently associated with all-cause mortality (HR: 3.26 [95% CI: 2.04-5.20], *P* < 0.001) (Figure 1, Supplemental Table 3), rehospitalization for HF (sHR: 2.08 [95% CI: 1.04-4.04], *P* = 0.038) (Figure 2, Supplemental Table 4), the composite endpoint of rehospitalization for HF and all-cause mortality (HR: 2.48 [95% CI: 1.64-3.74], *P* < 0.001) (Figure 3, Supplemental Table 5), and treatment futility (HR: 2.99 [95% CI: 1.76-5.04], *P* < 0.001) (Supplemental Table 6). Harrell's C-index and the likelihood ratio test demonstrated incremental prognostic value of HE4 ≥130 pmol/L over the nested model (diabetes, COPD, atrial fibrillation, CAD, LVEF <50%, and NT-ProBNP ratio >3) for all-cause mortality. The addition of HE4 ≥130 pmol/L improved the C-index from 0.63 (95% CI: 0.56-0.69) to 0.72 (95% CI: 0.67-0.78) and significantly enhanced model fit (likelihood ratio test, chi-square = 29.66, *P* < 0.001). Decision curve analysis demonstrated that the model with HE4 demonstrated a higher net benefit compared to the model without HE4 in the threshold probability range of 10% to 65%, indicating its added value in improving risk stratification and clinical decision-making within this range Supplemental Figure 4.

In subsequent multivariable Cox regression models adjusted for NT-ProBNP ratio ≥3 and STS score, a serum level of HE4 ≥130 pmol/L remained independently associated with a higher risk of all-cause mortality, the composite endpoint of rehospitalization for HF and all-cause mortality, and treatment futility (Supplemental Table 7).

A Kaplan-Meier analysis stratifying the cohort based on the NYHA functional class (I-II or III-IV) and HE4 serum levels (<130 or ≥130 pmol/L) is presented in Supplemental Figure 5. Patients with elevated HE4 serum levels had an increased rate of all-cause mortality, regardless of the NYHA functional class (log-rank *P* < 0.001).

### Association between HE4 and myocardial fibrosis

Among the 43 patients, 32/43 (74%) and 11/43 (26%) patients had a HE4 serum level <130 and ≥130 pmol/L, respectively (Supplemental Table 8). Patients with a HE4 serum level <130 pmol/L prior TAVI, patients with a higher HE4 serum level had higher median native T1 mapping values (1,278 [IQR: 1,239-1,280] ms vs 1,352 [IQR: 1,303-1,376] ms, *P* < 0.001) and numerically higher but not statistically significant rates of LGE positive (14 [60.9%] vs 6 [100%]; *P* = 0.065) and extracellular volume (25% [IQR: 21%-27%] vs 28.5% [IQR: 25%-31%]; *P* = 0.097) at 1 to 3 months following the procedure (Central Illustration, Supplemental Table 9). There was a modest but statistically significant correlation between HE4 serum levels and native T1 mapping on CMR (r = 0.42, *P* = 0.007) (Supplemental Figure 6). The optimal diagnostic threshold to identify diffuse myocardial fibrosis by native T1 mapping was 130 pmol/L (AUC: 0.69 [95% CI: 0.53-0.85], *P* = 0.038) (Supplemental Figure 7). In univariate logistic regression analysis, a serum level of HE4 ≥130 pmol/L was associated with diffuse myocardial fibrosis, as detected by native T1 mapping (OR: 13.07 [95% CI: 1.48-115.54], *P* = 0.021, Supplemental Table 10).

## Discussion

The main findings of the present study are 1) higher HE4 serum levels (≥130 pmol/L) are robustly associated with adverse clinical outcomes and treatment futility following TAVI; 2) higher HE4 serum levels are associated with the presence of myocardial fibrosis at 1 to 3 months following the procedure.

### HE4 as a promising blood biomarker for risk stratification in patients undergoing TAVI

HE4 is expressed in a variety of bodily organs, including the kidney, digestive tract, and respiratory system.^16^^,^^17^ HE4 is also known as a protease inhibitor that suppresses the activities of multiple proteases, such as serine proteases and matrix metalloproteinases.^18^ This secretory protein was originally used to diagnose ovarian cancer^30^ and has been shown to be associated with higher rates of rehospitalization and mortality in the HF population, as well as other cardiovascular diseases but has not yet been examined in the valvular heart disease population.^19^, ^20^, ^21^^,^^31^ To our knowledge, this is the first study to examine and demonstrate the association between HE4 serum levels with adverse clinical events in patients undergoing TAVI and assess its association with myocardial fibrosis. Herein, we report that high HE4 serum levels prior to TAVI are independently associated with higher rates of treatment futility, rehospitalization for HF, all-cause mortality, and are also associated with diffuse myocardial fibrosis.

Our findings are consistent with recent studies involving patients with HF, where higher HE4 serum levels were associated with increased HF severity and worse survival outcomes, regardless of comorbidities and LV function.^19^^,^^20^^,^^31^ In the present study, 43 (30.1%) patients with HE4 ≥130 pmol/L were oligosymptomatic and had increased risk of all-cause mortality, therefore confirming again the nonspecific value of symptoms in the context of AS, and the need for new parameters for risk stratification in such population.

Blood biomarkers can be obtained easily at low cost, and often demonstrate incremental prognostic value when compared to surgical risk scores.^13^^,^^15^ A previous study of Huang et al^32^ including 139 patients with chronic kidney disease and with 34% of patients suffering from acute HF demonstrated a strong correlation between NT-proBNP and HE4 serum levels (r = 0.65, *P* < 0.001). This observation was confirmed in the present study where a significant correlation was found between these 2 biomarkers (r = 0.51, *P* < 0.001). Interestingly, HE4 showed greater prognostic value to predict all-cause mortality vs NT-proBNP ratio or STS score. This superiority of HE4 may be explained by the fact that, as opposed to NT-proBNP, HE4 is not only a marker of cardiac damage but also of damage to other organs including kidneys and lungs as well as several types of cancer.^16^^,^^17^^,^^33^, ^34^, ^35^ HE4 demonstrated a higher net benefit compared to the model without HE4 in the threshold probability range of 10% to 65%, indicating its added value in improving risk stratification and clinical decision-making within this range. However, for threshold probabilities above 30%, the curves of both models overlapped, suggesting limited incremental value of HE4 in these cases. Overall, HE4 enhances clinical decision-making in a relevant range of thresholds, thereby supporting its inclusion as a complementary biomarker.

### HE4 as early marker of LV decompensation

According to current guidelines, left ventricular decompensation is typically defined by the apparition of symptoms and indicators of cardiac dysfunction such as a LVEF<50%, or markedly elevated (3-fold) BNP.^22^^,^^36^ Although myocardial fibrosis being a key pathological process leading to cardiac decompensation and worse outcomes, current guidelines do not propose this parameter as a trigger for intervention in patients with severe AS.^22^^,^^36^ The presence of focal fibrosis, as detected by LGE using CMR, is generally irreversible and associated with increased mortality following AVR,^28^^,^^37^ while diffuse fibrosis identified by native T1 mapping is, at least in part, reversible and allows identification of early LV decompensation.^38^ However, routine surveillance using CMR is challenging due to limited accessibility and high costs. In this context, blood biomarkers may be helpful to screen for myocardial fibrosis and identify patients who may require CMR to confirm and quantitate the extent of myocardial fibrosis.

HE4 has been demonstrated to be involved in both renal and myocardial fibrosis.^21^^,^^39^ In the present study, there was a modest, but significant correlation between serum levels of HE4 and diffuse fibrosis. In addition, there was a numerically higher but not statistically significant association between HE4 and replacement myocardial fibrosis as detected by LGE. These findings suggest that HE4 could potentially serve as a surrogate marker of myocardial fibrosis, aiding in the early detection of cardiac fibrosis and thus in decision-making for timing of intervention in asymptomatic patients with severe AS. Further analyses with larger cohorts are needed to confirm the diagnostic accuracy and prognostic value of this promising blood biomarker in patients undergoing TAVI.

### Clinical implications

On the one hand, we are in an era when the indication of TAVI is expanding to lower risk populations. On the other hand, to avoid unnecessary procedures and spare health care resources, it becomes a high priority to identify, prior to the procedure, the patients in whom TAVI has a high likelihood to be futile. The assessment of circulating HE4 may be useful in this regard. Higher HE4 levels may indeed represent a surrogate marker of diffuse and focal myocardial fibrosis (ie, cardiac damage) in the early stages but also damage to other organs such as renal and pulmonary damage. Both diffuse and focal myocardial fibrosis have been linked with worse prognosis.^40^, ^41^, ^42^, ^43^, ^44^ Hence, in patients referred for TAVI and exhibiting high levels of HE4, it may be useful to consider CMR in order to assess the extent of myocardial fibrosis, cardiac, and other organs damage and the ensuing risk of treatment futility. Patients with extensive focal fibrosis may not improve LV function following TAVI and are at high risk of treatment futility, whereas patients with mild to moderate diffuse myocardial fibrosis are more likely to improve their LV function and quality of life.

### Study limitations

The present study has several limitations. First, due to its observational nature, the presence of unrecognized confounders that can influence study conclusions cannot be excluded, although a robust adjustment that considers several baseline clinical characteristics was used to mitigate any potential bias. Second, it is a single-center, prospective, and observational study. Additionally, the contraindication for gadolinium administration in one-third of patients who underwent CMR limited further analyses regarding the association of HE4 serum levels and focal myocardial fibrosis, as detected by LGE. Moreover, as highlighted in previous studies, HE4 is not specific to AS and may be influenced by age and comorbidities such as renal failure, lung diseases, and ovarian cancer.^17^^,^^20^^,^^45^ Although this study demonstrates an association between HE4 and myocardial fibrosis as well as adverse clinical outcomes, further larger, multicenter studies are required to confirm the clinical utility of HE4 in identifying treatment futility and guiding therapeutic decision-making.

## Conclusions

In patients with severe AS who undergoing TAVI, elevated HE4 serum levels before the procedure are robustly associated with diffuse myocardial fibrosis and higher risk of treatment futility and adverse clinical events following the procedure. This novel biomarker may be useful to enhance risk stratification in patients undergoing TAVI and for therapeutic decision-making in asymptomatic patients with severe AS.

Perspectives **COMPETENCY IN MEDICAL KNOWLEDGE:** Higher HE4 serum levels (≥130 pmol/L) are associated with adverse clinical outcomes and treatment futility following TAVI, even in asymptomatic patients with severe AS. Elevated HE4 serum levels are also associated with myocardial fibrosis at 1 to 3 months following the procedure. This novel blood biomarker may be useful to enhance risk stratification in patients undergoing TAVI.

**TRANSLATIONAL OUTLOOK:** Further studies are needed to independently validate the diagnostic and prognostic value of HE4 in clinical practice. These preliminary results should be confirmed in larger cohorts.

## Funding support and author disclosures

Dr Pibarot has received a research grant from the 10.13039/501100000024Canadian Institutes of Health Research (grant #FDN-143225; Ottawa, Ontario, Canada). The study was supported by Roche Diagnostics International. Dr Clavel holds the Canada Research Chair on Females’s Cardiac Valvular Health from the Canadian Institutes of Health Research. Dr Bernard is supported by a doctoral scholarship from Canadian Institute of Health Research (CIHR). Dr Tastet was supported by a doctoral scholarship from Fonds de Recherche du Québec: Santé (FRQS). Dr Arsenault is supported by a senior scholar award from the FRQS. Dr Rodés-Cabau holds the Famille Jacques Larivière Chair in Structural Heart Disease. Dr Pibarot holds the Canada Research Chair in Valvular Heart Diseases, Canadian Institutes of Health Research and has received funding from Roche Diagnostic, Edwards Lifesciences, and Medtronic for echocardiography CoreLab analyses with no personal compensation. Dr Clavel has research contracts or grants with Edwards Lifesciences, Medtronic, Pi-Cardia, and Icon, with no personal compensation. All other authors have reported that they have no relationships relevant to the contents of this paper to disclose.
