# Supplementary material for: Human Epididymis Protein 4 in Transcatheter Aortic Valve Implantation: Diagnostic and Prognostic Value
Source: JACC Adv. 2025 Apr 25;4(5):101722. doi: 10.1016/j.jacadv.2025.101722 (PMC12103096; doi:10.1016/j.jacadv.2025.101722)
Supplement: Supplementary data [file mmc1.docx]

**Supplemental Table 1.** Procedural Data

|  | **All Patients**  n = 362 | **HE4 <130 pmol/L**  n = 219 (60.5%) | **HE4 ≥130 pmol/L**  n = 143 (39.5%) | **P Value** |
| --- | --- | --- | --- | --- |
| **Procedural approach, n (%)** |  |  |  | 0.129 |
| Transfemoral | 243 (66.9) | 156 (71.2) | 86 (60.1) |  |
| Transcarotid | 95 (26.5) | 52 (23.7) | 43 (30.1) |  |
| Transapical | 18 (5) | 9 (0.9) | 9 (6.3) |  |
| Transaortic | 6 (1.7) | 2 (0.9) | 4 (2.8) |  |
| **Prosthesis type, n (%)** |  |  |  | 0.979 |
| Sapien 3 | 225 (62.2) | 136 (62.1) | 89 (62.2) |  |
| Evolut R | 85 (23.5) | 45 (20.5) | 40 (28.0) |  |
| Others | 52 (14.3) | 38 (17.4) | 14 (9.8) |  |
| THV size (≤23 mm), n (%) | 82 (22.6) | 39 (27.7) | 29 (25) | 0.020 |
| Pre-dilatation, n (%) | 37 (10.3) | 52 (23.7) | 8 (5.6) | **0.018** |
| Post-dilatation, n (%) | 49 (13.6) | 29 (13.3) | 20 (14.1) | 0.833 |
| Valve in valve, n (%) | 52 (14.4) | 27 (12.3) | 25 (17.6) | 0.158 |
| Procedural success, n (%) | 354 (98.6) | 212 (98.1) | 114 (99.3) | 0.365 |
| **Legends:** THV, Transcatheter heart valve. | | | | |

**Supplemental Table 2.** Short- and Mid- term Outcomes Following TAVI

|  | **All Patients**  n = 362 | **HE4 <130 pmol/L**  n = 219 (60.5%) | **HE4 ≥130 pmol/L**  n = 143 (39.5%) | **p Value** |
| --- | --- | --- | --- | --- |
| **Short-term outcomes** |  |  |  |  |
| THV malposition or embolization, n (%) | 0 | 0 | 0 | **-** |
| More than one THV, n (%) | 3 (0.8) | 2 (1.1) | 1 (0.6) | 0.562 |
| MI or coronary obstruction, n (%) | 7 (1.9) | 3 (1.4) | 4 (2.8) | 0.235 |
| Stroke/TIA, n (%) | 12 (3.3) | 7 (3.2) | 5 (3.5) | 0.866 |
| Dialysis, n (%) | 29 (8.1) | 8 (3.7) | 21 (15.1) | **<0.001** |
| New onset of atrial fibrillation, n (%) | 22 (6.1) | 9 (4.1) | 13 (9.2) | **0.050** |
| Need of pacemaker, n (%) | 61 (16.9) | 33 (15.1) | 28 (19.7) | 0.325 |
| Hospital stays, days | 6 (4-9) | 5 (3-8) | 7 (4-14) | **<0.001** |
| 30-days all-cause mortality, n (%) | 8 (2.2) | 2 (0.9) | 6 (4.2) | **0.038** |
| 1-year all-cause mortality, n (%) | 35 (9.5) | 6 (2.7) | 29 (20.3) | **<0.001** |
| **Mid-term outcomes** |  |  |  |  |
| All-cause mortality, n (%) | 99 (27.3) | 33 (15.1) | 66 (46.1) | **<0.001** |
| Cardiovascular death, n (%) | 31 (8.5) | 10 (4.6) | 21 (14.7) | **0.001** |
| Cardiac death, n (%) | 23 (6.4) | 9 (4.1) | 14 (9.8) | **0.030** |
| Rehospitalization for HF, n (%) | 34 (9.4) | 13 (5.8) | 21 (14.7) | **0.005** |
| **Legends:** CHF, congestive heart failure; IQR, interquartile range; MI, myocardial infarction; THV, transcatheter heart valve; TIA, transient ischemic attack. | | | | |

**Supplemental Table 3.** Uni- and Multivariable Cox Regression Analysis of Baseline Factors Associated with All-Cause Mortality

|  | **Univariate Analysis** |  | **Multivariable Analysis** |  |
| --- | --- | --- | --- | --- |
|  | **HR [95% CI]** | **P value** | **HR [95% CI]** | **P value** |
| **Clinical Data** |  | | | |
| Age, years | 1.00 [0.98 – 1.03] | 0.543 |  |  |
| Men | 1.16 [0.77 – 1.74] | 0.461 |  |  |
| Body mass index, kg/m² | 0.81 [0.54 – 1.20] | 0.810 |  |  |
| Hypertension | 1.46 [0.77 – 2.76] | 0.243 |  |  |
| Dyslipidemia | 0.89 [0.53 – 1.51] | 0.668 |  |  |
| Diabetes mellitus | 1.53 [1.02 – 2.29] | **0.039** | 1.11 [0.72 – 1.72] | 0.609 |
| Active cancer | 1.23 [0.74 – 2.04] | 0.407 |  |  |
| In remission cancer | 1.12 [0.35 – 3.56] | 0.847 |  |  |
| CHF | 1.66 [1.11 – 2.47] | **0.012** |  |  |
| Previous MI | 1.02 [0.51 – 2.03] | 0.950 |  |  |
| COPD | 1.73 [1.11 – 2.69] | **0.014** | 1.73 [1.11 – 2.71] | **0.015** |
| History of AF | 1.50 [1.00 – 2.23] | **0.046** | 0.93 [0.61 – 1.43] | 0.769 |
| CAD | 2.10 [1.31 – 3.35] | **0.002** | 2.17 [1.32 – 3.56] | **0.002** |
| Renal failure | 0.72 [0.48 – 1.08] | 0.122 |  |  |
| EuroSCORE II | 1.01 [0.98 – 1.04] | 0.282 |  |  |
| STS score | 1.02 [1.00 – 1.05] | **0.019** |  |  |
| NYHA functional class ≥ III | 1.29 [0.83 – 2.01] | 0.248 |  |  |
| **Laboratory Data** |  |  |  |  |
| NT-ProBNP, pg/ml | 1.00 [1.00 – 1.00] | **0.003** |  |  |
| NT-ProBNP, ratio ≥3* | 2.27 [1.50 – 2.44] | **<0.001** | 1.64 [1.01 – 2.66] | **0.043** |
| HE4≥130 pmol/L | 3.45 [2.14 – 5.57] | **<0.001** | 3.26 [2.04 – 5.20] | **<0.001** |
| **Echocardiographic Data** |  | | | |
| LVEF, % | 0.82 [0.96 – 0.99] | **0.016** |  |  |
| LVEF <50 % | 1.61 [1.04 – 2.49] | **0.031** | 0.84 [0.50 – 1.39] | 0.503 |
| MG, mmHg | 0.99 [0.98 – 1.00] | 0.170 |  |  |
| EOAi, cm^2^/m^2^ | 0.80 [0.07 – 1.00] | 0.854 |  |  |
| SVi, mL/m^2^ | 0.96 [0.93 – 0.99] | **0.031** |  |  |
| SVi <35 mL/m^2^ | 1.37 [0.80 – 2.34] | 0.243 |  |  |
| AR ≥ moderate | 0.77 [0.43 – 1.38] | 0.383 |  |  |
| MR ≥ moderate | 1.05 [0.65 – 1.68] | 0.830 |  |  |
| TR ≥ moderate | 1.88 [0.98 – 3.66] | 0.055 |  |  |
| PAPs ≥ 50 mmHg | 0.74 [0.49 – 1.14] | 0.747 |  |  |
| **Legends:** As Table 1 and 2. CI, confidence interval; HR, hazard ratio. CK-MB; creatine kinase myocardial band, HE4, human epididymis protein 4, NT-proBNP, N-terminal B-type natriuretic peptide. * Compared with normal BNP level. | | | | |

**Supplemental Table 4.** Fine and Gray Uni- and Multivariable Analysis of Baseline Factors Associated with Rehospitalization for HF

|  | **Univariate Analysis** | |  | **Multivariable Analysis** |  |
| --- | --- | --- | --- | --- | --- |
|  | **sHR [95% CI]** | | **P value** | **sHR [95% CI]** | **P value** |
| **Clinical Data** |  |  | | | |
| Age, years | 1.04 [1.00 – 1.08] | | **0.032** |  |  |
| Men | 0.63 [0.31 – 1.29] | | 0.210 |  |  |
| Body mass index, kg/m² | 1.00 [0.97 – 1.04] | | 0.786 |  |  |
| Hypertension | 4.78 [0.64 – 35.41] | | 0.125 |  |  |
| Dyslipidemia | 6.45 [0.88 – 47.04] | | 0.066 |  |  |
| Diabetes mellitus | 1.65 [0.85 – 3.23] | | 0.141 |  |  |
| Active cancer | 1.22 [0.69 – 2.16] | | 0.491 |  |  |
| CHF | 1.86 [0.95 – 3.63] | | 0.068 |  |  |
| Previous MI | 0.58 [0.14 – 2.39] | | 0.447 |  |  |
| COPD | 1.51 [0.72 – 3.15] | | 0.276 |  |  |
| History of AF | 2.44 [1.25 – 4.79] | | **0.009** | 1.99 [0.99 – 3.97] | 0.052 |
| CAD | 1.18 [0.58 – 2.38] | | 0.646 |  |  |
| Renal failure | 1.67 [1.12 – 2.49] | | **0.012** |  |  |
| EuroSCORE II | 1.03 [1.00 – 1.07] | | **0.030** |  |  |
| STS score | 1.07 [1.05 – 1.09] | | **<0.001** |  |  |
| NYHA functional class ≥ III | 1.13 [0.55 – 2.32] | | 0.732 |  |  |
| **Laboratory Data** |  | |  |  |  |
| NT-ProBNP, pg/ml | 1.00 [1.00 – 1.00] | | **<0.001** |  |  |
| NT-ProBNP, ratio ≥3* | 2.51 [1.25 – 5.07] | | **<0.001** | 1.79 [0.89 – 3.60] | 0.103 |
| HE4≥130 pmol/L | 2.62 [1.31 – 5.25] | | **0.007** | 2.08 [1.04 – 4.04] | **0.038** |
| **Echocardiographic Data** |  | |  |  |  |
| LVEF, % | 0.95 [0.94 – 0.97] | | **<0.001** |  |  |
| LVEF <50 % | 4.75 [2.43 – 9.30] | | **<0.001** |  |  |
| MG, mmHg | 0.97 [0.94 – 0.99] | | **0.026** |  |  |
| EOAi, cm^2^/m^2^ | 0.18 [0.01 – 2.16] | | 0.178 |  |  |
| SVi, mL/m^2^ | 0.95 [0.88 – 1.02] | | 0.164 |  |  |
| SVi <35 mL/m^2^ | 1.35 [0.47 – 3.89] | | 0.581 |  |  |
| AR ≥ moderate | 1.36 [0.59 – 3.14] | | 0.465 |  |  |
| MR ≥ moderate | 2.41 [1.22 – 4.74] | | **0.011** |  |  |
| TR ≥ moderate | 0.96 [0.33 – 2.82] | | 0.946 |  |  |
| PAPs ≥ 50 mmHg | 1.02 [0.51 – 2.03] | | 0.954 |  |  |
| **Legends:** As Online Table Table 3. | | | | | |

**Supplemental Table 5.** Uni- and Multivariable Cox Regression Analysis of Baseline Factors Associated with Composite Endpoint

|  | **Univariate Analysis** |  | **Multivariable Analysis** |  |
| --- | --- | --- | --- | --- |
|  | **HR [95% CI]** | **P value** | **HR [95% CI]** | **P value** |
| **Clinical Data** |  | | | |
| Age, years | 1.01 [0.98 – 1.03] | 0.358 |  |  |
| Men | 1.25 [0.85 – 1.83] | 0.245 |  |  |
| Body mass index, kg/m² | 1.00 [0.97 – 1.03] | 0.921 |  |  |
| Hypertension | 1.79 [0.92 – 3.46] | 0.082 |  |  |
| Dyslipidemia | 1.12 [0.66 – 1.91] | 0.653 |  |  |
| Diabetes mellitus | 1.51 [1.04 – 2.21] | **0.030** | 1.24 [0.83 – 1.86] | 0.280 |
| Active cancer | 1.50 [0.95 – 2.37] | 0.075 |  |  |
| In remission cancer | 1.16 [0.42 – 3.20] | 0.761 |  |  |
| CHF | 1.63 [1.12 – 2.37] | **0.010** |  |  |
| Previous MI | 0.96 [0.80 – 1.94] | 0.902 |  |  |
| COPD | 1.49 [0.98 – 2.25] | 0.060 |  |  |
| History of AF | 1.59 [1.10 – 2.31] | **0.014** | 1.25 [0.85 – 1.84] | 0.241 |
| CAD | 1.72 [1.13 – 2.60] | **0.010** | 1.64 [1.07 – 2.50] | **0.021** |
| Renal failure | 1.39 [0.95 – 2.04] | 0.082 |  |  |
| EuroSCORE II | 1.03 [1.00 – 1.05] | **0.007** |  |  |
| STS score | 1.06 [1.04 – 1.09] | **<0.001** |  |  |
| NYHA functional class ≥ III | 1.36 [0.90 – 2.05] | 0.143 |  |  |
| **Laboratory Data** |  |  |  |  |
| NT-ProBNP, pg/ml | 1.00 [1.00 – 1.00] | **<0.001** |  |  |
| NT-ProBNP, ratio ≥3* | 2.33 [1.58 – 3.43] | **<0.001** | 1.52 [0.96 – 2.40] | 0.069 |
| HE4≥130 pmol/L | 3.15 [2.15 – 4.62] | **<0.001** | 2.48 [1.64 – 3.74] | **<0.001** |
| **Echocardiographic Data** |  |  |  |  |
| LVEF, % | 0.97 [0.96 – 0.99] | **0.001** |  |  |
| LVEF <50 % | 2.06 [1.39 – 2.02] | **<0.001** | 1.27 [0.80 – 2.01] | 0.301 |
| MG, mmHg | 0.99 [0.97 – 1.00] | 0.077 |  |  |
| EOAi, cm^2^/m^2^ | 3.24 [0.38 – 28.12] | 0.277 |  |  |
| SVi, mL/m^2^ | 0.97 [0.94 – 1.00] | 0.084 |  |  |
| SVi <35 mL/m^2^ | 1.36 [0.82 – 2.27] | 0.225 |  |  |
| AR ≥ moderate | 0.86 [0.80 – 1.46] | 0.576 |  |  |
| MR ≥ moderate | 1.19 [0.78 – 1.82] | 0.411 |  |  |
| TR ≥ moderate | 1.79 [0.98 – 3.25] | 0.055 |  |  |
| PAPs ≥ 50 mmHg | 0.85 [0.27 – 1.25] | 0.415 |  |  |
| **Legends:** As Online Table 3. | | | | |

**Supplemental Table 6**. Uni- and Multivariable Cox Regression Analysis of Baseline Factors Associated with Treatment Futility.

|  | **Univariate Analysis** |  | **Multivariable Analysis** |  |
| --- | --- | --- | --- | --- |
|  | **OR [95% CI]** | **P value** | **OR [95% CI]** | **P value** |
| **Clinical Data** |  | | | |
| Age, years | 1.00 [0.97 – 1.03] | 0.710 |  |  |
| Men | 1.08 [0.77 – 1.63] | 0.736 |  |  |
| Body mass index, kg/m² | 1.00 [0.97 – 1.04] | 0.654 |  |  |
| Hypertension | 3.30 [1.03 – 10.52] | **0.043** | 2.95 [0.92 – 9.46] | 0.068 |
| Dyslipidemia | 1.66 [0.69 – 3.05] | 0.313 |  |  |
| Diabetes mellitus | 1.80 [1.13 – 2.89] | **0.013** | 1.49 [0.92 – 2.42] | 0.100 |
| Active cancer | 1.17 [0.64 – 2.15] | 0.595 |  |  |
| In remission cancer | 0.98 [0.23 – 4.01] | 0.975 |  |  |
| CHF | 1.21 [0.74 – 1.98] | 0.431 |  |  |
| Previous MI | 0.90 [0.39 – 2.09] | 0.822 |  |  |
| COPD | 1.69 [1.02 – 2.80] | **0.041** | 1.55 [0.93 – 2.58] | 0.089 |
| History of AF | 1.11 [0.68 – 1.81] | 0.652 |  |  |
| CAD | 1.27 [0.78 – 2.09] | 0.329 |  |  |
| Renal failure | 1.44 [0.89 – 2.32] | 0.134 |  |  |
| EuroSCORE II | 1.02 [0.99 – 1.05] | 0.129 |  |  |
| STS score | 1.04 [1.01 – 1.04] | **0.011** |  |  |
| NYHA functional class ≥ III | 1.29 [0.77 – 2.18] | 0.324 |  |  |
| **Laboratory Data** |  |  |  |  |
| NT-ProBNP, pg/ml | 1.00 [1.00 – 1.00] | **0.012** |  |  |
| NT-ProBNP, ratio ≥3* | 2.02 [1.25 – 3.28] | **0.004** | 1.28 [0.74 – 2.22] | 0.366 |
| HE4≥130 pmol/L | 3.59 [2.18 – 5.91] | **<0.001** | 2.99 [1.76 – 5.04] | **<0.001** |
| **Echocardiographic Data** |  |  |  |  |
| LVEF, % | 0.96 [0.93 – 0.98] | **0.007** |  |  |
| LVEF <50 % | 1.81 [1.09 – 2.98] | **0.020** | 1.28 [0.73 – 2.25] | 0.383 |
| MG, mmHg | 0.99 [0.97 – 1.00] | 0.268 |  |  |
| EOAi, cm^2^/m^2^ | 1.35 [0.56 – 3.21] | 0.498 |  |  |
| SVi, mL/m^2^ | 1.00 [0.96 – 1.04] | 0.988 |  |  |
| SVi <35 mL/m^2^ | 1.08 [0.59 – 2.17] | 0.817 |  |  |
| AR ≥ moderate | 1.04 [0.55 – 1.04] | 0.898 |  |  |
| MR ≥ moderate | 1.22 [0.72 – 2.08] | 0.444 |  |  |
| TR ≥ moderate | 1.18 [0.56 – 2.16] | 0.656 |  |  |
| PAPs ≥ 50 mmHg | 1.15 [0.71 – 1.86] | 0.545 |  |  |
| **Legends:** As Online Table 3. | | | | |

**Supplemental Table 7.** Multivariable Cox Regression Analyses of the Association Between HE4 Serum Levels and All-Cause Mortality, Composite Outcomes, and Treatment Futility.

|  | **Multivariable analysis** |  |
| --- | --- | --- |
|  | **HR [95% CI]** | **P value** |
| **All-cause Mortality** |  |  |
| HE4≥130 pmol/L | 2.97 [1.84 – 4.78] | **<0.001** |
| **All-cause Mortality and Rehospitalization for HF** |  |  |
| HE4≥130 pmol/L | 2.76 [1.52 – 5.01] | **<0.001** |
| **All-cause Mortality, NHYA ≥III or Rehospitalization for HF at 1 year** |  |  |
| HE4≥130 pmol/L | 2.98 [1.72 – 5.17] | **<0.001** |
| **Legends:** Adjusted for STS score, NT-ProBNP ratio≥3 and a serum level of HE4 ≥130 pmol/l.  CI, confidence interval; HE4, human epididymis protein 4; HF, heart failure; HR, hazard ratio; NHYA, New York Heart Association; NT-proBNP, N-terminal B-type natriuretic peptide. | | |

**Supplemental Table 8.** Baseline Characteristics of CMR Patients According to HE4 Serum Level

| **Clinical Data** | **All patients**  **N= 43** | | **HE4 <130 pmol/L**  **N=32 (74%)** | **HE4≥130 pmol/L**  **N=11 (26%)** | **P value** |
| --- | --- | --- | --- | --- | --- |
| Age, years | | 77.4±1.6 | 76.7±1.8 | 79.4±3.5 | 0.401 |
| Men sex, n (%) | | 28 (65.1) | 19 (59.4) | 9 (81.8) | 0.178 |
| Body mass index, kg/m^2^ | | 27.11±0.83 | 27.8±0.97 | 26.1±1.50 | 0.138 |
| Hypertension, n (%) | | 38 (88.4) | 27 (84.4) | 11 (100) | 0.163 |
| Dyslipidemia, n (%) | | 38 (88.4) | 27 (84.4) | 11 (100) | 0.163 |
| Smoking history, n (%) | | 16 (37.2) | 10 (31.25) | 6 (54.5) | 0.215 |
| Diabetes mellitus, n (%) | | 15 (34.9) | 11 (34.4) | 4 (36.4) | 0.905 |
| Atrial fibrillation, n (%) | | 12 (27.9) | 7 (22.6) | 5 (45.5) | 0.149 |
| Prior LBBB, n (%) | | 5 (11.6) | 3 (9.4) | 2 (18.2) | 0.733 |
| CAD, n (%) | | 28 (65.1) | 22 (68.8) | 6 (54.5) | 0.394 |
| History of CABG, n (%) | | 15 (34.9) | 11 (34.4) | 4 (36.4) | 0.905 |
| History of PCI, n (%) | | 16 (37.2) | 13 (40.6) | 3 (27.3) | 0.429 |
| Cancer, n (%) | | 13 (30.2) | 8 (25) | 5 (45.5) | 0.098 |
| CHF, n (%) | | 11 (25.6) | 4 (12.5) | 7 (63.6) | **0.001** |
| Previous MI, n (%) | | 4 (9.3) | 3 (9.7) | 1 (9.1) | 0.955 |
| Cerebrovascular disease, n (%) | | 1 (2.3) | 0 (0) | 1 (9.1) | 0.084 |
| History of TIA, n (%) | | 2 (4.7) | 1 (3.1) | 1 (9.1) | 0.418 |
| Peripheral vascular disease, n (%) | | 10 (23.3) | 8 (25) | 2 (18.2) | 0.644 |
| COPD, n (%) | | 10 (23.3) | 6 (18.8) | 4 (36.4) | 0.233 |
| Renal failure, n (%) | | 15 (34.9) | 8 (25) | 7 (63.6) | **0.020** |
| NYHA functional ≥ III, n (%) | | 24 (55.8) | 18 (56.3) | 6 (54.5) | 0.922 |
| STS score | | 4.60±0.67 | 3.35±0.36 | 7.84±2 | **0.022** |
| EuroSCORE II | | 5.33±0.84 | 4.53±0.85 | 7.45±1.79 | 0.059 |
| **Laboratory Data** | |  |  |  |  |
| HE4, pmol/L | | 100.40 (68.01-133-80) | 88.73 (63.00-107.00) | 196.30 (157.00-365.20) | - |
| Hemoglobin, g/L | | 126.00 (111.00-134.00) | 129.00 (113.00-134.75) | 112.00 (101.00-127.00) | **0.034** |
| Nt-ProBNP, pg/ml | | 940.10 (245.60-2643.00) | 492.50 (263.20-2141.65) | 2470.00 (565.70-7414.10) | **0.032** |
| Creatinine, mmol/l | | 83.00 (69.00-106.00) | 74.00 (62.75-89.50) | 135.00 (96.00-226.00) | **<0.001** |
| eGFr, ml/min | | 61.72(41.00-80.07) | 70.93 (53.75-91.55) | 33.12 (22.14-55.59 | **<0.001** |
| **Legends:** As Table 1 and 2. | | | | | |

**Supplemental Table 9.** CMR Characteristics According to HE4 Serum Level

| **CMR Characteristics** | **All patients**  **N= 43** | **HE4 <130 pmol/L**  **N=32 (74%)** | **HE4≥130 pmol/L**  **N=11 (26%)** |  |
| --- | --- | --- | --- | --- |
| \| LVEDVi (mL/m^2^) \| 75.15±3.78 \| 72.58±3.93 \| 80.99±8.65 \| 0.332 \| \| --- \| --- \| --- \| --- \| --- \| \| LV SVi (mL/m^2^) \| 41.98±1.52 \| 41.81±1.45 \| 42.37±3.87 \| 0.427 \| \| LVEF (%) \| 57.51±1.83 \| 59.26±1.90 \| 53.54±4.04 \| 0.247 \| \| LVMi (g/m^2^) \| 67.21±2.74 \| 65.20±3.04 \| 71.77±5.70 \| 0.210 \| \| RVEDVi (mL/m^2^) \| 78.33±3.15 \| 74.05±2.68 \| 88.06±7.81 \| 0.080 \| \| RV SVi (mL/m^2^) \| 42.32±1.33 \| 41.55±1.43 \| 44.05±2.96 \| 0.498 \| \| RVEF (%) \| 55.30±1.51 \| 56.53±1.31 \| 52.50±3.95 \| 0.612 \| \| LAVi (mL/m^2^) \| 44.76±3.30 \| 43.60±4.22 \| 47.71±4.78 \| 0.414 \| \| LGE, n (%) (29/43) \| 20 (69) \| 14 (60.9) \| 6 (100) \| 0.065 \| \| T1 Mapping, (ms) \| 1280 (1253- 1357) \| 1278 (1239 – 1280) \| 1352 (1303- 1376) \| **0.001** \| \| T1 Mapping≥ 1280 ms, n (%) \| 23 (56.1) \| 13 (43.3) \| 10 (90.9) \| **0.007** \| \| ECV (%) \| 26 (21- 28) \| 25 (21- 27) \| 28.5 (25-31) \| 0.097 \| \| T1 Mapping≥1280 ms + ECV≥26% (33/43) \| 8 (24.2) \| 3 (12) \| 5 (62.5) \| **0.001** \| \| ECV>26% \| 16 (53.3) \| 9 (39.1) \| 5 (71.4) \| 0.134 \| \| **Legends:** CMR, cardiac magnetic resonance; ECV, extracellular volume fraction; HE4, human epididymis protein 4; LAVi, indexed left atrial volume; LGE, late gadolinium enhancement; LVEDVi, indexed left ventricular end-diastolic volume; LVSVi, indexed left ventricular stroke volume; LVEF, left ventricular ejection fraction. LVMi, left ventricular mass indexed; RVEDVi, indexed right ventricular end-diastolic volume; RVSVi, indexed right ventricular stroke volume; RVEF, right ventricular ejection fraction. \| \| \| \| \| | | | | |

**Supplemental Table 10.** Univariate Analysis of Factors Associated with Diffuse Myocardial Fibrosis as Assessed by Native T1 Mapping

|  | **Univariate analysis** |  |
| --- | --- | --- |
|  | **OR [95% CI]** | **P value** |
| **Clinical Data** |  |  |
| Age, years | 1.02 [0.96 – 1.08] | 0.440 |
| Men | 0.68 [0.18 – 2.52] | 0.572 |
| Body mass index, kg/m² | 0.93 [0.82 – 1.06] | 0.326 |
| Hypertension | 2.10 [0.31 – 14.15] | 0.446 |
| Dyslipidemia | 6.28 [0.63 – 62.16] | 0.116 |
| Diabetes mellitus | 1.19 [0.33 – 4.28] | 0.787 |
| Smoking history | 1.10 [0.24 – 4.85] | 0.900 |
| Cancer | 3.26 [0.57 – 18.46] | 0.180 |
| CHF | 2.66 [0.59 – 12.05] | 0.202 |
| Previous MI | 1.52 [0.12 – 18.32] | 0.740 |
| COPD | 1.23 [0.29 – 5.26] | 0.775 |
| History of AF | 12.30 [1.38 – 109.09] | **0.024** |
| CAD | 1.28 [0.35 – 4.66] | 0.702 |
| Renal failure | 2.69 [0.67 – 10.74] | 0.161 |
| Creatinine clearence (Cockcroft) | 6.00 [0.65 – 55.30] | 0.114 |
| EuroSCORE II | 1.08 [0.93 – 1.25] | 0.275 |
| STS score | 1.38 [0.96 – 1.98] | 0.079 |
| NYHA functional class ≥ III | 1.55 [0.44 – 5.41] | 0.487 |
| **Echocardiography Data** |  |  |
| Left ventricular ejection fraction, % | 0.99 [0.93 – 1.06] | 0.936 |
| MG, mmHg | 0.97 [0.93 – 1.01] | 0.157 |
| EOA, cm^2^ | 8.22 [0.23 - 286.31] | 0.245 |
| **Cardiac Magnetic Resonance Data** |  |  |
| Indexed left ventricular end-diastolic volume, mL/m^2^ | 1.04 [1.00 – 1.08] | **0.045** |
| LVEF<50% | 2.18 [0.47 – 10.05] | 0.314 |
| SVi, mL/m^2^ | 1.05 [0.98 – 1.11] | 0.109 |
| Left ventricular mass index, g/m^2^ | 1.02 [0.99 – 1.06] | 0.091 |
| Indexed left atrial volume, mL/m^2^ | 1.01 [0.98- 1.05] | 0.331 |
| Indexed right ventricular end-diastolic volume, mL/m^2^ | 1.05 [0.99- 1.10] | 0.074 |
| Right ventricular ejection fraction, % | 0.97 [0.90 – 1.05] | 0.557 |
| **Laboratory Data** |  |  |
| HE4 ≥130 pmol/L | 13.07 [1.48–115.54] | **0.021** |
| Nt-ProBNP, pg/ml | 1.00 [1.00–1.01] | 0.09 |
| NT-ProBNP, ratio ≥3* | 4.87 [1.27–18.64] | **0.021** |
| Hemoglobine, gr/L | 0.98 [0.94–1.01] | 0.356 |
| **Legends:** As Table 1 and 2. | | |

**Supplemental Figure 1.** Prognostic Threshold of HE4 Associated with All-Cause Mortality

**
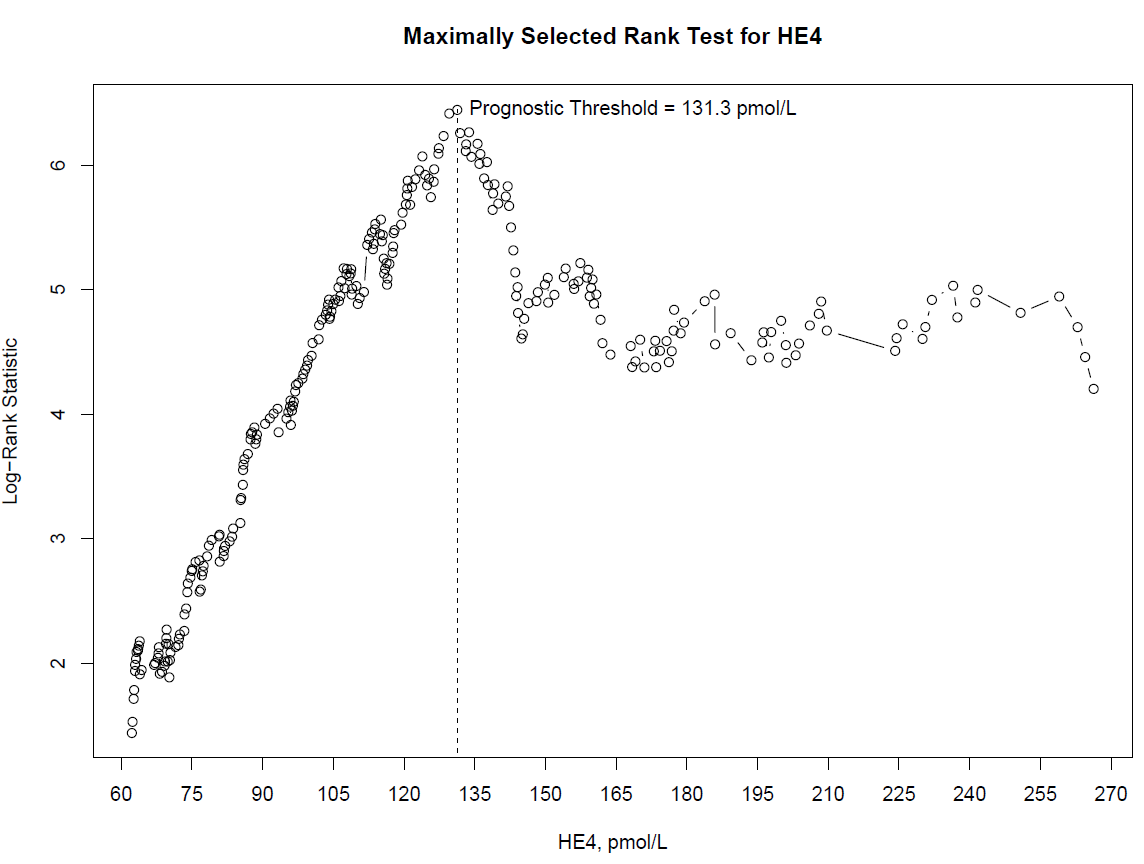
**

**Supplemental Figure 2.** Prevalence of Symptoms According to HE4 Serum Level


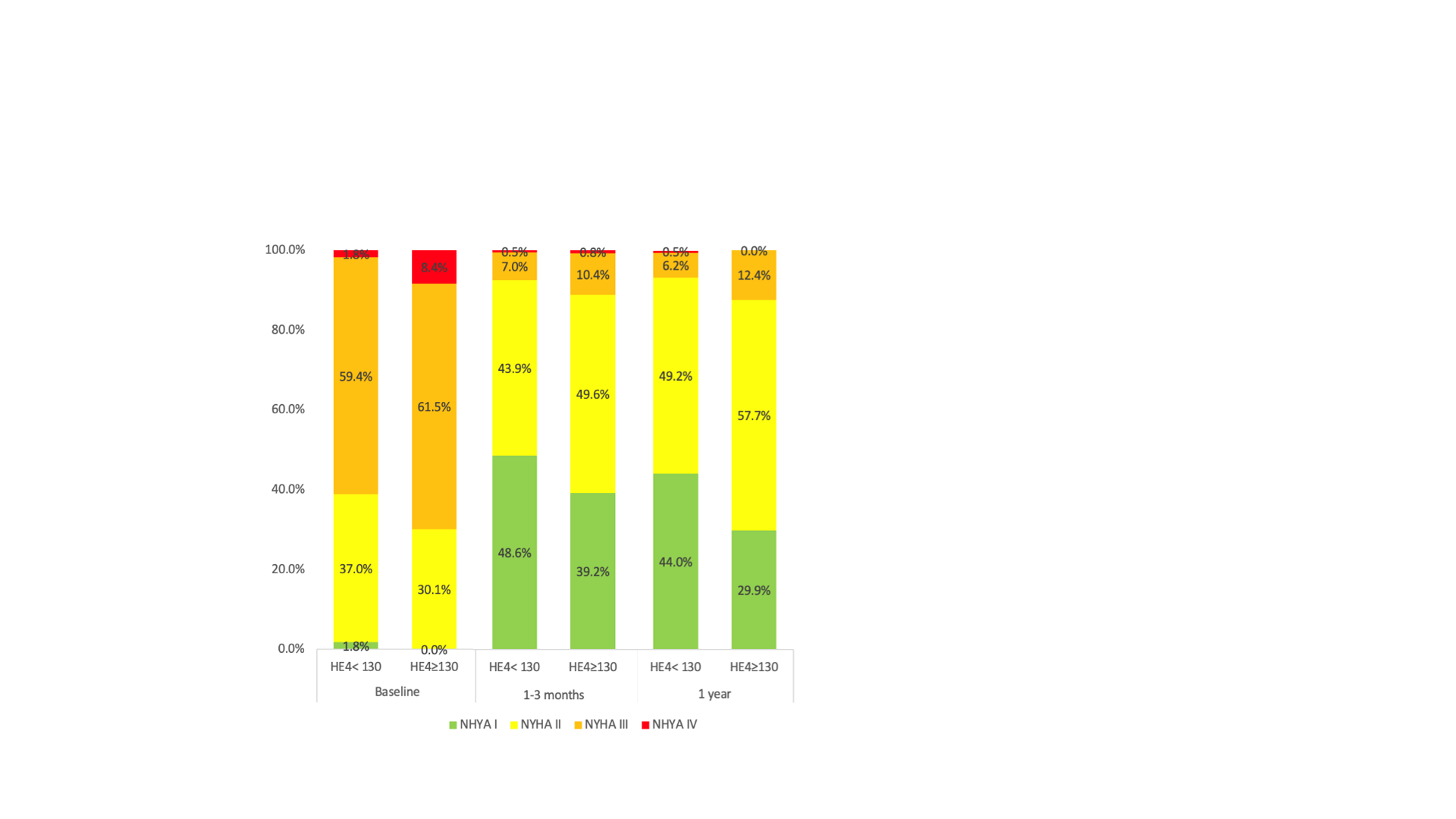


**Legends**: HE4, human epididymis protein 4; NHYA, New York Heart Association.

**Supplemental Figure 3.** Prognostic Value of HE4, STS Score and NT-proBNP to Predict All-Cause Mortality


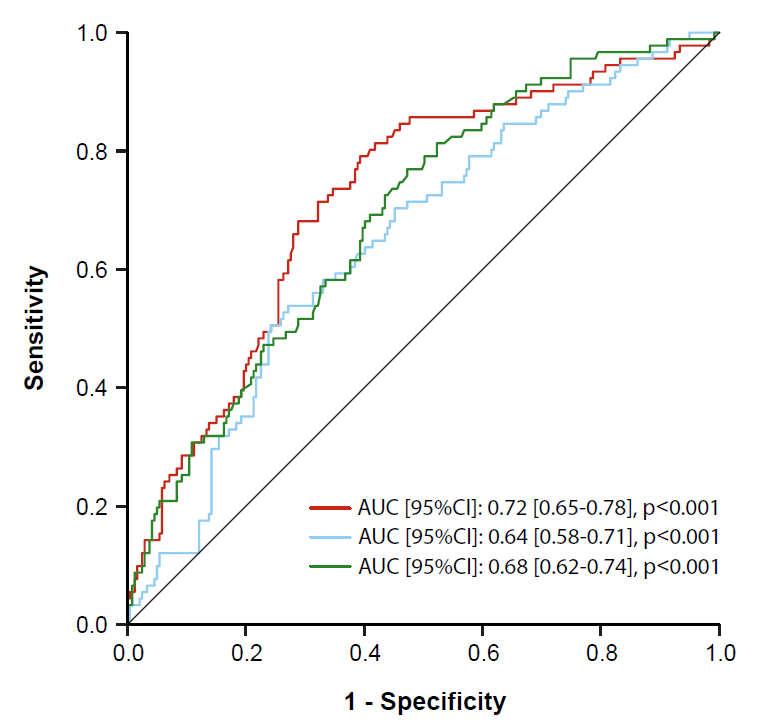


**Legends:** ROC curve analysis assessing the prognostic value of HE4 (red line), STS score (green line) and NT-proBNP (blue line) to predict all-cause mortality. AUC, area under the curve; CI, confidence interval; HE4, human epididymis protein 4; Nt-ProBNP, N-terminal B-type natriuretic peptide; STS score, Society Thoracic Surgeons; ROC, receiver operating curve.

**Supplemental Figure 4.** Decision Curve Analysis (DCA) to assess the clinical utility of HE4≥130 plasma level.

**
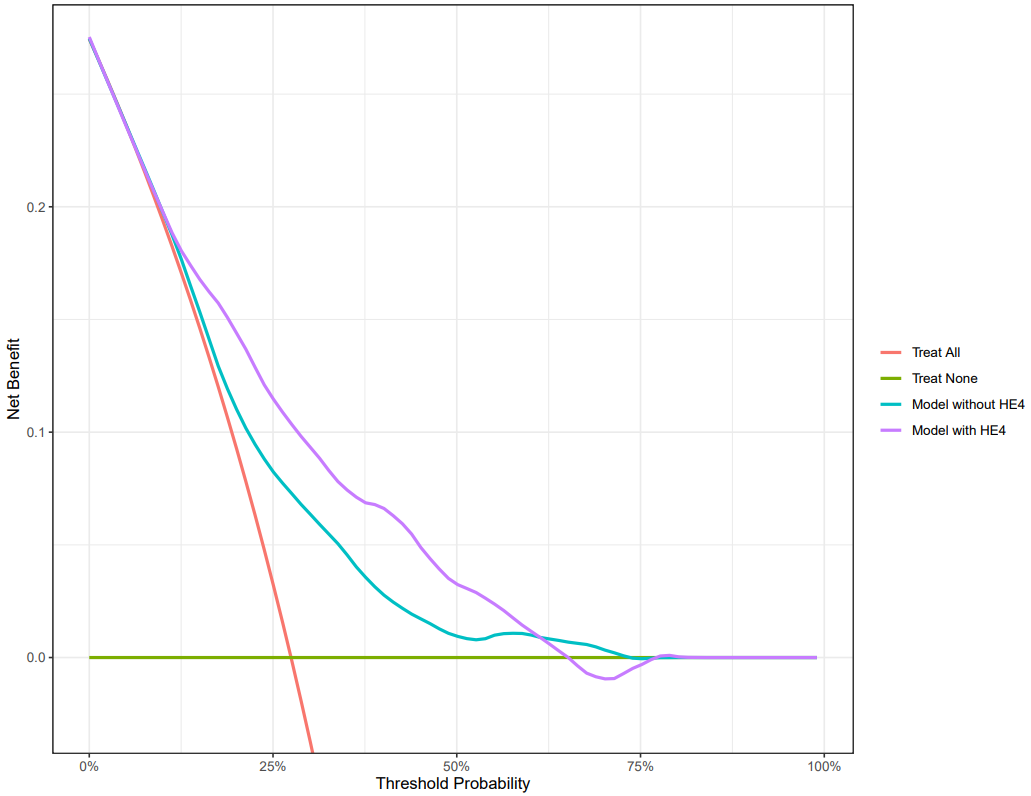
**

**Legends**: DCA, Decision Curve Analysis to assess the clinical utility with model of HE4 (purple line); model of HE4 (turquoise line); treat all (orange line); treat none (green line); HE4, human epididymis protein.

**Supplemental Figure 5.** HE4 Prognostic Value According to NYHA Functional Classification and HE4 Serum Level


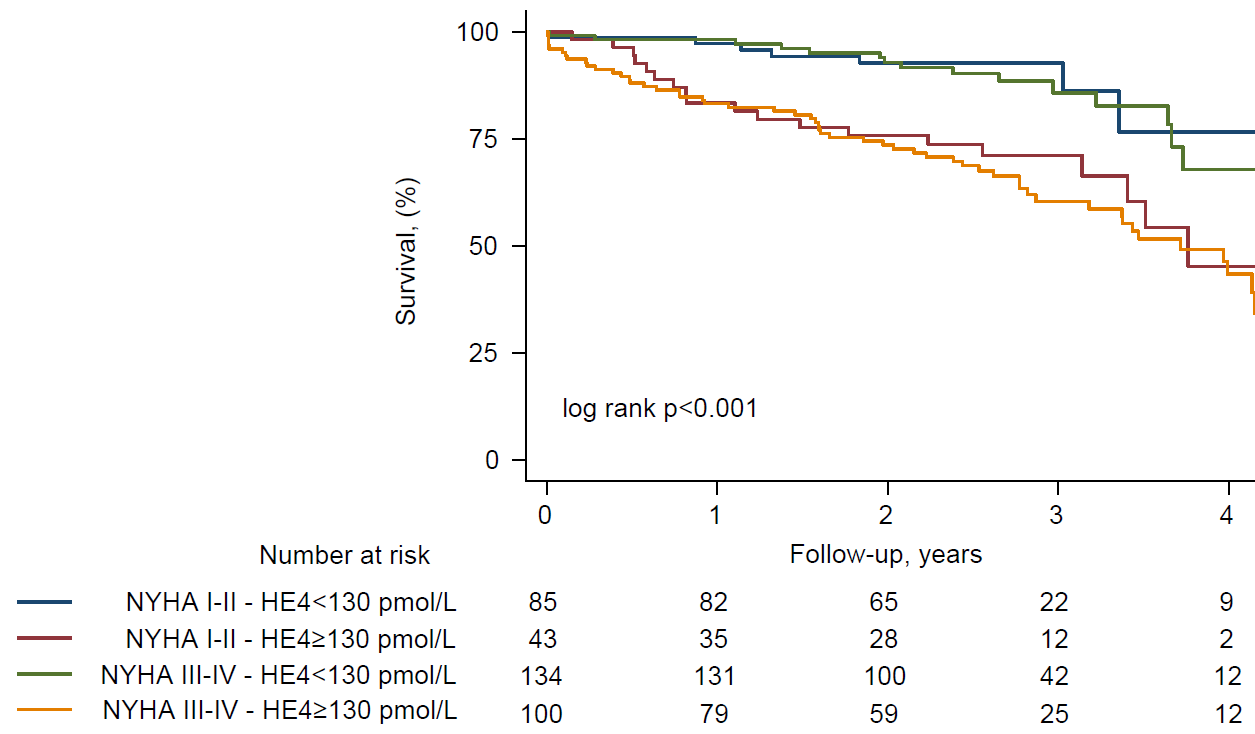


**Legends**: NHYA, New York Heart Association; HE4, human epididymis protein 4.

**Supplemental Figure 6.** Correlation Between HE4 Serum Level and Native T1 Mapping (Diffuse Myocardial Fibrosis) Values by CMR


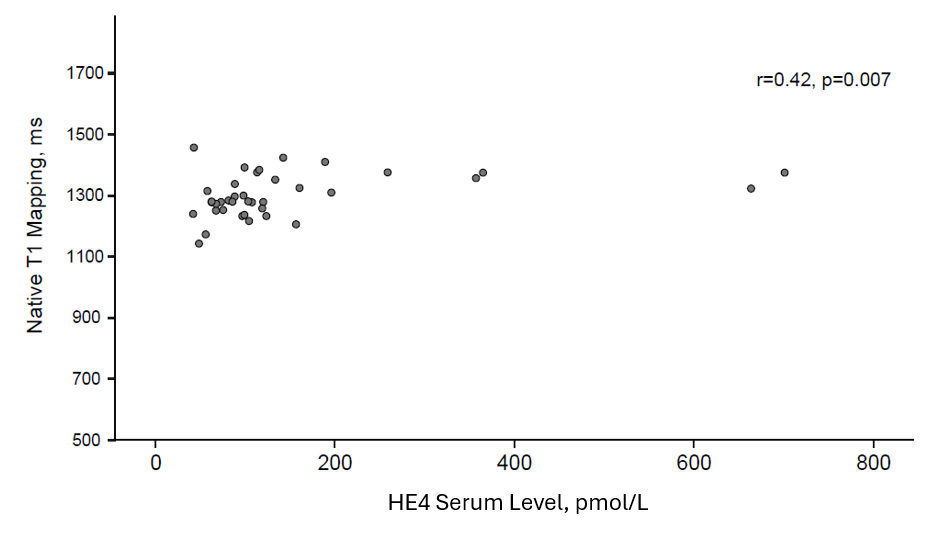


**Legends:** Correlation between HE4 serum level and native T1 mapping by CMR. HE4, human epididymis protein 4 (pmol/L) and native T1 mapping (ms)

**Supplemental Figure 7.** Diagnostic Value of HE4 Serum Level to Predict Myocardial Fibrosis as Assessed by Native T1 Mapping


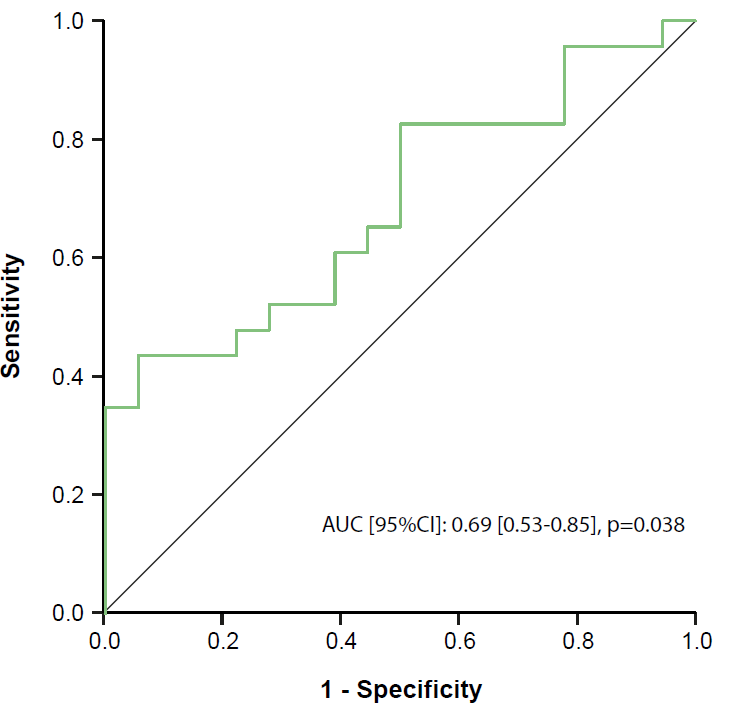


**Legends:** AUC, area under the curve; CI, confidence interval; HE4, human epididymis protein 4.
